# Molecular Epidemiology of HIV-1 Subtypes in India: Origin and Evolutionary History of the Predominant Subtype C

**DOI:** 10.1371/journal.pone.0039819

**Published:** 2012-06-29

**Authors:** Ujjwal Neogi, Irene Bontell, Anita Shet, Ayesha De Costa, Soham Gupta, Vishal Diwan, Ranbir S. Laishram, Ajay Wanchu, Udaykumar Ranga, Akhil C. Banerjea, Anders Sönnerborg

**Affiliations:** 1 Unit of Infectious Diseases, Department of Medicine Huddinge, Karolinska Institutet, Stockholm, Sweden; 2 Division of Clinical Virology, Department of Microbiology, St. John’s Medical College, Bangalore, Karnataka, India; 3 Division of Global Health (IHCAR), Nobels Väg 9, Karolinska Institutet, Stockholm, Sweden; 4 Department of Pediatrics, St. John’s Medical College Hospital, Bangalore, Karnataka, India; 5 Department of Public Health and Environment, R. D. Gardi Medical College, Ujjain, Madhya Pradesh, India; 6 Department of Pediatrics, Regional Institute of Medical Science, Imphal, Manipur, India; 7 Departnemt of Internal Medicine, Postgraduate Institute of Medical Education and Research, Chandigarh, India; 8 Division of Arthritis and Rheumatic Disease, Oregon Health and Science University, Portland Oregon, United States of America; 9 Molecular Biology and Genetics Unit, Jawaharlal Nehru Centre for Advanced Scientific Research, Bangalore, Karnataka, India; 10 Virology-II, National Institute of Immunology, New Delhi, India; 11 Division of Clinical Virology, Department of Laboratory Medicine, Karolinska Institutet, Karolinska University Hospital, Stockholm, Sweden; University of Florida, United States of America

## Abstract

**Background:**

India has the third largest HIV-1 epidemic with 2.4 million infected individuals. Molecular epidemiological analysis has identified the predominance of HIV-1 subtype C (HIV-1C). However, the previous reports have been limited by sample size, and uneven geographical distribution. The introduction of HIV-1C in India remains uncertain due to this lack of structured studies. To fill the gap, we characterised the distribution pattern of HIV-1 subtypes in India based on data collection from nationwide clinical cohorts between 2007 and 2011. We also reconstructed the time to the most recent common ancestor (tMRCA) of the predominant HIV-1C strains.

**Methodology/Principal Findings:**

Blood samples were collected from 168 HIV-1 seropositive subjects from 7 different states. HIV-1 subtypes were determined using two or three genes, *gag, pol,* and *env* using several methods. Bayesian coalescent-based approach was used to reconstruct the time of introduction and population growth patterns of the Indian HIV-1C. For the first time, a high prevalence (10%) of unique recombinant forms (BC and A1C) was observed when two or three genes were used instead of one gene (p<0.01; p = 0.02, respectively). The tMRCA of Indian HIV-1C was estimated using the three viral genes, ranged from 1967 (*gag*) to 1974 (*env*). *Pol-*gene analysis was considered to provide the most reliable estimate [1971, (95% CI: 1965–1976)]. The population growth pattern revealed an initial slow growth phase in the mid-1970s, an exponential phase through the 1980s, and a stationary phase since the early 1990s.

**Conclusions/Significance:**

The Indian HIV-1C epidemic originated around 40 years ago from a single or few genetically related African lineages, and since then largely evolved independently. The effective population size in the country has been broadly stable since the 1990s. The evolving viral epidemic, as indicated by the increase of recombinant strains, warrants a need for continued molecular surveillance to guide efficient disease intervention strategies.

## Introduction

India has a burden of 2.4 million people infected with human immunodeficiency virus type 1 (HIV-1) making it the third largest HIV-1 epidemic in the world after South Africa and Nigeria (National AIDS Control Organization, Annual Report 2011, http://nacoonline.org.). HIV-1 in India was first identified among female sex workers in Tamil Nadu in 1986 [1]. Molecular epidemiological analysis has identified the predominant subtype as subtype C (HIV-1C), irrespective of route of transmission [2]–[5]. Most of the reports have been limited by sample size, uneven geographical distribution and the number of viral genes studied [6]–[8]. A recent study categorized all reported HIV sequences from India into two periods (2000 to 2003 and 2004 to 2007) and identified a dominance of HIV-1C, which constituted 96.5% and 97.8% of all infections, respectively, although inherent biases of small sample size and simplified subtyping methods were acknowledged as study limitations [9].

The reconstruction of the time to most recent common ancestor (tMRCA) of HIV-1C in Africa has been dated back to the early 1950s [10], [11]. Studies have also been conducted to identify the origin and tMRCA of subtype C in Malawi [12], southern America [13], [14], Zimbabwe [15], Ethiopia [16] and the UK [17]. A recent report dated the tMRCA of HIV-1C in India back to the mid- to late of the 1970s [18], [19]. Although the estimate is indicative, the assessment was based on a relatively small number of sequences, retrieved from a secondary database, with little knowledge of the geographic origins. These factors introduce an element of uncertainty to the estimate, which could be overcome using a larger sample of sequences with known origins from different parts of the country. Furthermore, studies based on a single gene analysis may suffer from a limitation in that the rate of evolution of the viral genes may differ significantly due to the variable selection pressure exerted by host factors [10]. As a consequence the actual date of viral introduction may be underestimated [14].

The present study is a comprehensive investigation of the molecular epidemiology of the HIV-1 subtypes circulating in India based on a large number of clinical samples representing different geographic regions using three different viral genes *gag*, *pol* and *env*. To trace the date of introduction of HIV-1C in India, we generated datasets representing seven states of the country; Punjab and Haryana (northern region), Manipur (north-eastern region), Madhya Pradesh (central region), Karnataka, Andhra Pradesh and Tamil Nadu (southern region). Additionally, sequences representing the state of Maharashtra (the western region) were downloaded from the databases. Our study thus represents the first nationwide study to examine the molecular distribution of the circulating HIV-1 subtypes in India and to trace the tMRCA of the predominant subtype C strains.

## Results

A total of 168 samples were used representing the northern (n = 27), north-eastern (n = 15), central (n = 25) and southern (n = 101) regions of India. The *gag, pol* and *env* gene segments were amplified from 164 (97.6%), 120 (71.4%) and 158 (94%) samples, respectively. The three genes *gag/pol/env* were amplified from 107 patients; *gag/pol* from 116, *gag/env* from 155 and *pol/env* from 110 individuals. The patient clinical and demographic characteristics were as follows: mean age was 36 years (SD±9) and 55.4% (93/168) were male. The predominant route of transmission was heterosexual in 89.9% (151/168) of the subjects, while the remaining were intravenous drug users (8.9%; 15/168) and perinatal transmission (1.2%; 2/168). Median CD4 count (available for 141 patients) was 213 cells/mm^3^ (IQR: 120–339) and mean viral load (available for 80 patients) was 5.5 Log_10_ copies/mL (SD±0.6). Mean known duration of sero-positivity available for 115 patients was 12 months (Range 0–130 months). All the patients were therapy naïve.

### HIV-1 Subtyping

**Figure 1:**
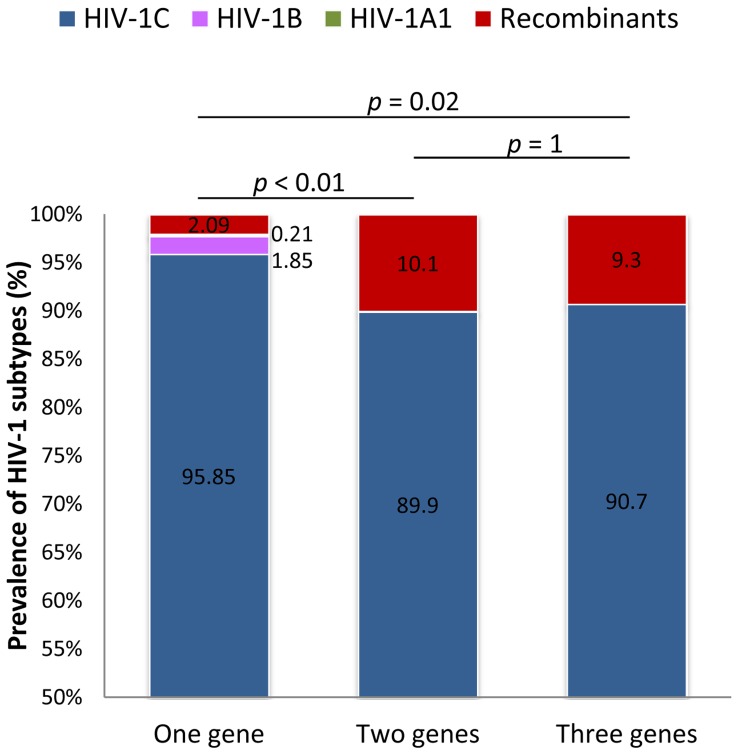
Prevalence of HIV-1 subtypes and recombinant forms in India based on one gene, two and three genes. The *p* values are presented along with the values for the predominant HIV-1C and recombinants. Subtyping was carried out as described in the methods section. For sequences that indicated an event of recombination, bootscan analysis was performed in Simplot version 3.5.1 using 100 nucleotide window size and 20 nucleotide step size to map the precise breakpoint. The recombination event was further confirmed by region-specific phylogenetic analysis using ML tree in MEGA 5.

Subtyping analysis confirmed the predominance of HIV-1C strains in India. The prevalence of recombinants depended on whether one or more genes were analyzed. When a single gene was used for the subtype determination the mean proportion of HIV-1C, B and A1 were 95.9%, 1.9% and 0.2%, respectively, while the recombinants constituted 2.1%. A significant increase was observed in the prevalence of recombinant strains when two genes or three genes were used for subtype determination (10.1%; 17/168, p<0.01; 9.4%; 10/107, p = 0.02) (Figure 1).

**Figure 2:**
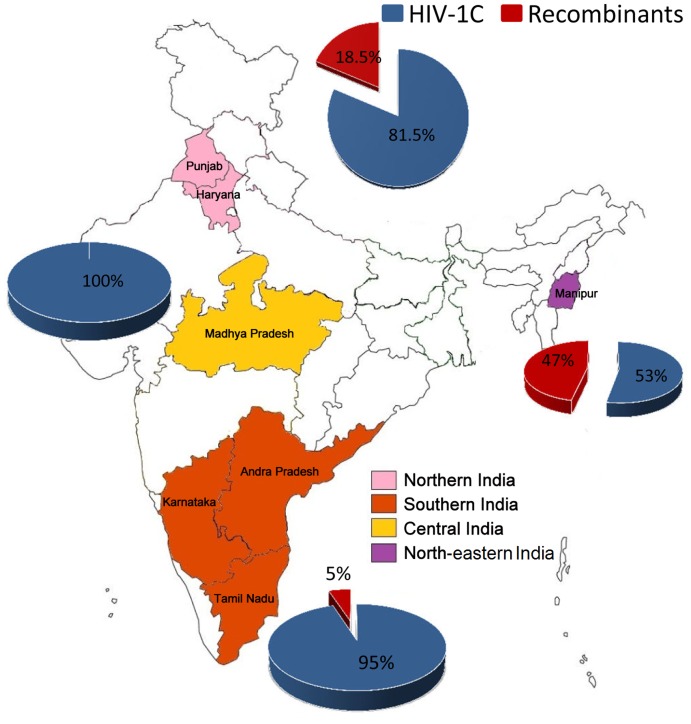
Distribution of HIV-1 subtypes and recombinants in the clinical cohorts based on two genes. The regions [southern (Karnataka, Tamil Nadu and Andhra Pradesh), northern (Punjab and Haryana), north-eastern (Manipur) and central (Madhya Pradesh)] from where the samples were collected are shown in colors. The pie chart depicts the percentage of subtypes and recombinant strains in respective regions.

Among the four geographical regions examined, a high proportion of recombinant strains was identified in north-eastern (46.7%; 7/15) and northern (18.5%; 5/27) India (Figure 2). While in central India, all strains were identified as HIV-1C and in southern India 5.0% of the strains (5/101) were identified to be recombinant. Of the recombinant strains, 6.0% (10/168) were recombinants of subtypes B and C, whereas 4.2% (7/168) were recombinants of A1 and C. Furthermore, these 17 recombinant strains represented a large magnitude of genetic diversity - at least five different B-C and three different A1-C recombinants (Table 1). All the identified HIV-1B and A1 strains in the single gene analysis turned out to be recombinant strains when two or three viral genes were taken into considerations. The genetic configuration of all recombinant strains has been depicted (Table 1). It has to be noted that out of the 17 recombinant strains, four BC recombinant stains showed recombination breakpoint in the *pol* gene while three A1C recombinant strains showed recombination breakpoint in the *env* gene. Also, the number of degenerate bases were low in all samples, which were classified as recombinant strains, thus eliminating the possibility of dual infection. The nearest sequence analysis using BLAST identified that the subtype B segments in the BC recombinant strains as derived most likely from China and Thailand while the A1 segment in A1C originated from eastern Africa (Kenya, Uganda and Tanzania). These results were further confirmed by the maximum likelihood (ML) tree.

**Table 1:** Genetic make-up of recombinant clinical strains.

PID	*gag*			*pol*			*env*		Nearest Strains (non-C subtype region)	Recombinant*
	RIP		REGA	RIP		REGA	RIP	REGA		
08NIIPGI01^a^	B		B	Not amplified			C	C	Thailand	URF_BC
08NIIPGI12^a^	B		B	Not amplified			C	C	China	URF_BC
08NIIPGI14^a^	C		C	Not amplified			A1, C	C	Kenya	URF_A1C
08NIIPGI19^a^	B		B	Not amplified			C	C	China	URF_BC
09NIIPGI47^a^	C		C	Not amplified			A1	*NA*	Uganda	URF_A1C
MNP01^b^	C		C	B, C	*NA*		C	C	Thailand	URF_BC
MNP05^b^	C		C	B	*NA*		C	C	China	URF_BC
MNP07^b^	C		C	B, C	*NA*		C	C	China	URF_BC
MNP08^b^	C		C	B, C	*NA*		C	C	China	URF_BC
MNP09^b^	B		B	B	B		C	C	China	URF_BC
MNP11^b^	C		C	B, C	*NA*		B	B	China	URF_BC
MNP15^b^	C		C	B	B		B, C	*NA*	China/Thailand	URF_BC
08JNC05^c^	C		C	Not amplified			A1,C	*NA*	Kenya	URF_A1C
09SJ08^c^	C		C	A1	A1		C	C	Cyprus	URF_A1C
09SJ56^c^	Not amplified			C	C		A1, C	*NA*	Kenya	URF_A1C
10SJ02^c^	C	C		C	C		A1	*NA*	Iran	URF_A1C
11SJ74^c^	C	C		Not amplified			A1	A1	Tanzania	URF_A1C

a Samples from northern India, ^b^North-eastern India and ^c^Southern India. The recombinants were identified based on atleast two of the three viral genes, *gag*, *pol* and *env*. Recombination Identification Program ver 3.0 (RIP) and REGA subtyping tool ver 2 (REGA) were used. Recombinant strains were further confirmed by bootscan analysis in Simplot version 3.5.1 using 100 nucleotide window size and 20 nucleotide step size to map the precise breakpoint and region-specific phylogenetic analysis using ML tree in MEGA 5. NA: Not Assigned.

### Date of Origin of the Predominant HIV-1C Strains in India

**Figure 3:**
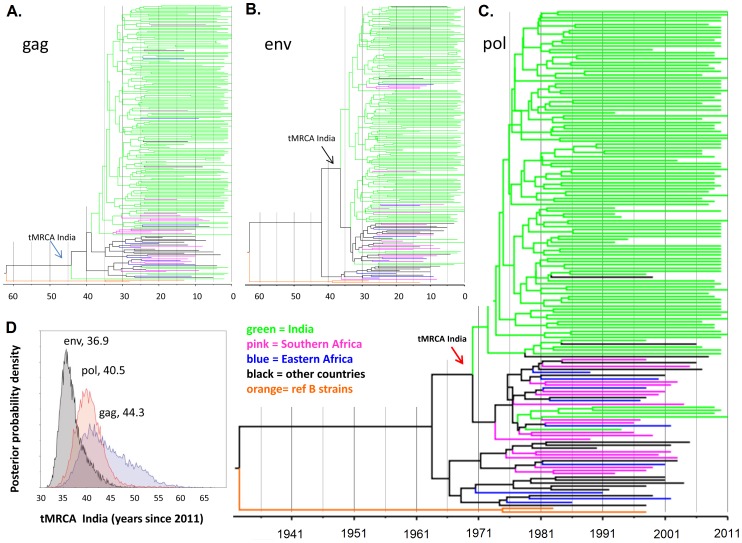
Annotated phylogenetic trees for the *gag*- (Panel A), *env* (Panel B) and *pol*- (Panel C) genes. Phylogenetic trees were constructed using the Indian HIV-1 subtype C from clinical samples taken from different parts of India (shown in green), including southern (Karnataka, Tamil Nadu and Andhra Pradesh), northern (Punjab and Haryana), north-eastern (Manipur) and central (Madhya Pradesh) regions of India plus data base retrieved reference sequences from western India and other countries, and two out-group subtype B strains. The Indian tMRCA included in this study were shown with an arrow. *Pol-*gene analysis was considered to provide the most reliable estimate of tMRCA (shown with a red arrow) for Indian HIV-1C, indicating around 1971 (95% CI: 1965–1976)]. **Posterior probability plots showing the tMRCA estimates of Indian HIV-1C (Panel D):** The posterior probability density plots for the three genes (*env*, *pol* and *gag*) point to a tMRCA for Indian HIV-1C from 36.9 (*env*), 40.5 (*pol*) and 44.3 (*gag*) years ago, with an overall mean for the combined graph at 40.6 years ago before 2011, that is in 1970.4 (95% CI: 1960.3–1978.1).

The tMRCA estimates using three viral genes ranged from year 1967 (*gag*) (95% CI: 1957–1975) to 1974 (95% CI: 1968–1978) (*env*), with the estimate for the *po*l-gene falling in between at 1971 (95% CI: 1965–1976) (Figure 3). The overall median and mean values for the three genes were found to be 1971 and 1970, respectively. The tMRCA analysis showed a good convergence and high effective sample size (ESS) values (>200) for most parameters of all three genes. For *gag* and *env,* however, a small number of Indian strains fell outside of the main Indian clade in the final tree. Since the outlier viral strains were also included in the taxon set for the Indian tMRCA calculations, it resulted in poor convergence and low ESS (<200) of this parameter. This problem, however, was not manifested in the *pol*-gene analysis. Therefore the *pol*-gene analysis is likely to provide the most reliable estimate for the India C tMRCA (1971), which agreed well with the overall mean and median values.

### The Geographical Source of HIV-1C in India

Our data points towards a first introduction of HIV-1 in India around 40 years ago and at that time, subtype C had recently emerged and the intra-subtype divergence seen today had not yet arisen. It was not possible to determine from which country the first introduction took place, as the main Indian clade of the *pol*-tree are equidistant to all strains in the neighboring clade. The relative lack of intermingling between Indian and African strains suggest that the main Indian HIV-1C epidemic has largely evolved independently over the last four decades, and that later transmission events between these regions have only caused a minor contribution to the overall prevalence in the Indian states included in this study.

### Phylogenetic Analysis and the Migration Pattern of HIV-1 Subtypes in India

Phylogenetic analyses of the three viral genes showed that a large majority of all Indian HIV-1C strains included in the analysis (91.3% for *gag*, 95.7% for *env* and 95.5% for *pol*) clustered together in the main Indian clades (Figure 3). This shows that most infections are acquired within the country, but detailed regional transmission patterns could not be discerned. An intense admixing was observed among the northern, western, central and southern Indian strains. The *pol* phylogenetic tree identified a small clade mainly consisting of the strains from western, central and north-eastern regions of the country, indicating movement of strains among these regions (data not shown).

### Population Growth Dynamics

**Figure 4:**
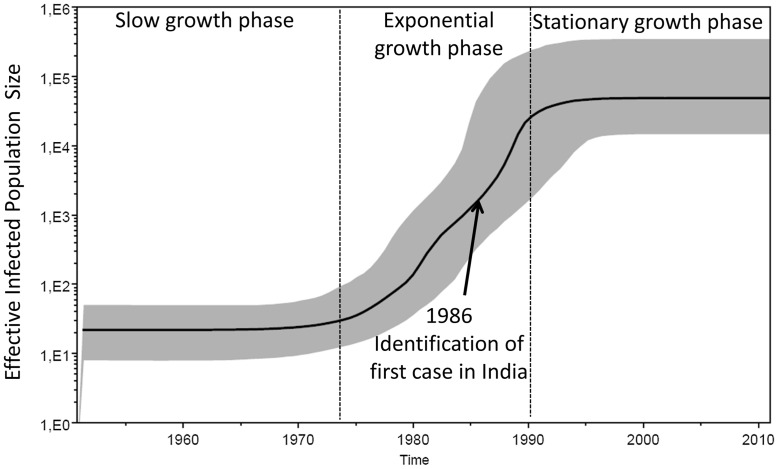
Bayesian skyline plot representing estimates of the effective number of infections through time. The demographic history from the pol BSP identified three epidemic growth phases divided by the vertical lines; an initial slow growth followed by an exponential phase which slowed down to a stationary phase around 1990. The identification of first HIV case in India is indicated by an arrow.

The Bayesian skyline plot (BSP) is used to draw an inference of the estimation of the effective population size (the number of infected individuals contributing to the spread of the disease) over time directly from the sequence data [20]. The demographic history from the *pol* BSP identified three epidemic growth phases (Figure 4), an initial slow growth phase until the mid1970s followed by an exponential growth phase till the late 1980 and early 1990s followed by a stationary phase, approaching the present time. A similar pattern was observed for *env* and *gag* BSP.

## Discussion

The present study reports for the first time the molecular epidemiology of the Indian HIV-1 epidemic using sequences of three structural genes (*gag*, *pol* and *env*) derived from multiple clinical cohorts across the country. Based on this high quality data set, we found a high prevalence of recombinant strains in India Also, our analysis suggests that the most recent common ancestor of the HIV-1C epidemic in India is dated between 1967 and 1974, more precisely in 1971, approximately five years earlier than previous estimates [18], [19].

All of the previous studies of the molecular epidemiology of the Indian HIV-1 epidemic have used a few geographically localized samples and a single viral gene [5]–[8], [21], [22]. A recent such study of global trends in the molecular epidemiology of HIV-1 identified 1.06% recombinant strains (CRFs and URFs) in India between 2000 and 2007 [9]. Our own observations were comparable for the period between 2007 and 2011 when a single viral gene was used in the analysis. However, when two or three viral genes were included, a higher proportion of HIV-1 recombinant strains were found than has been reported earlier [22]–[26]. The mosaic structure in our recombinants indicates that URFs might have been formed in the local epidemic due to migration of strains. Some of these URFs are likely to mature into CRFs as they continue to circulate among the populations.

In our study, URFs were more prevalent in these northern and north-eastern regions of India. This outcome, however, must be interpreted with caution given that the numbers of strains analysed from these two regions were relatively small. Previous reports from northern [22] and north-eastern [26] regions of India demonstrated relatedness to the subtype B segments from China and Southeast Asian countries, mainly Thailand. In addition, it has been reported that the Indian subtype C was one of the parental strains of CRF07_BC and CRF08_BC [18]. Thus, the data from our analysis and the previous studies collectively suggest an expansion of the HIV-1 URFs in the northern and north-eastern regions of India. Cross border networking among the intravenous drug users may be the driving force of the spread of recombination in these regions. Furthermore, our data indicate that the transmission of the subtype A1 segment of the A1C recombinants in India most likely occurred from eastern Africa. Continual migration between Africa, especially eastern and southern, and India, for business and migrant labour has been going on for centuries, and this well established trade route may explain the spread.

The tMRCA of HIV-1C in Africa has been dated into 1950s. Our estimates of the tMRCA of Indian HIV-1C to be between 1967 and 1974 allude to the presence of HIV-1C in Indian nearly two decades prior to its detection in 1986 [1]. The time of introduction of HIV-1C in India is somewhat later than that of Ethiopia (1965) [16] and Zimbabwe (early 1970s) [15], but precedes that in Brazil (early 1980s) [13] and the United Kingdom (1980s) [17]. This would indicate that HIV-1C was introduced in India at an early stage of the global subtype C epidemic and that it is likely to be the oldest HIV-1C epidemic outside Africa.

The population dynamics described in this report indicates the growth of the effective infected population size in three phases. The data corresponds well with the HIV-1 estimates in India, which indicate a stable or reverse HIV prevalence between 2002 and 2009 (National AIDS Control Organization, Annual Report 2011, http://nacoonline.org.). Our molecular data are also in line with the success of the strategic plan for the HIV prevention, the National AIDS Control Program (NACP) launched by Govt. of India in the 1990s. Targeted interventions through NACP –I-III to high risk group populations and the scale up of this program coincides with the stabilization of the epidemic as observed in our BSP.

Our study has some limitations. First, the depth of the sampling is relatively low given the HIV-1 estimates in the country. In order to more accurately analyze the spread of HIV-1C within the subcontinent, a further increase of the number of samples from the other locations would be needed. Second, the present analysis was restricted to mainly heterosexual transmission and only a few samples from intravenous drug users and perinatal transmission. Third, Bayesian coalescent method has some limitations to conclude the population demography as the deleterious mutations may result in overestimating the tMRCA. Fourth, we have a large number of samples from southern India compared to the other regions. However a technical merit of our study is the large data set with known clinical, demographical, sampling date and geographic origin. The multiple gene analysis for HIV-1 subtyping also minimizes the use of the subtype C segments of recombinant strains in the dataset to estimate the tMRCA.

In conclusion, our study identified a significant increase in the prevalence of recombinant strains (URFs) in India by the application of robust subtyping methods. The introduction of HIV-1C into India was dated back to around 1971, and we found that the epidemic has been stable for over a decade. Our results indicate that HIV-1C was likely to have been introduced into India at an early stage of the global HIV-1 epidemic and that India harbors one of the oldest HIV-1C epidemics worldwide outside Africa. As the depth of sampling (the proportion of available sequences to infections) is still very low in India the resulting diversified epidemiology may pose serious challenges to the development of an effective vaccine that would be applicable in the country. Ongoing country wide molecular surveillance of HIV-1 is likely to contribute towards a better understanding of the epidemiology in this region.

## Materials and Methods

### Study Population

Blood samples were obtained between 2007 and 2011 from HIV-1 infected seropositive subjects (n = 168) hailing from different regions of India including northern (Punjab and Haryana), southern (Karnataka, Tamil Nadu and Andhra Pradesh), north-eastern (Manipur) and central (Madhya Pradesh) regions. Written informed consent was obtained**.** Genomic DNA was extracted from whole blood using QIAamp Blood DNA kit (Qiagen, Germany) and stored at −80°C until used.

### Polymerase Chain Reaction and Sequencing

HIV-1 p17 region *gag* (HXB2 position 790–1190), RT region of *pol* (HXB2 position 2598–3254) and C2V4 region of *env* (HXB2 position 7050–7550), respectively, were amplified by nested polymerase chain reaction (PCR) using iNtRON Taq Polymerase (Intron Biotech, South Korea) as described previously [5], [21], [27]. The following amplification conditions were common for all three genes were: 1 cycle at 95°C for 2 min, 3 cycles at 94°C for 1 min, 55°C for 1 min and 72°C for 3 min, followed by 32 cycles at 94°C for 1 min, 60°C for 1 min and 72°C for 3 min with a final extension at 72°C for 10 min. Two µl PCR product from the first round was used as template in the second round nested PCR, with 1 cycle at 94°C for 2 min, 3 cycles at 94°C for 1 min, 55°C for 30 sec and 72°C for 3 min, followed by 32 cycles at 94°C for 1 min, 60°C for 1 min and 72°C for 1 min with a final extension at 72°C for 5 min. Bidirectional sequencing was carried out using the second round PCR primers.

### Genetic Subtyping of the Viral Strains

Reference sequences (2010) of different subtypes (n = 170) were downloaded from HIV-1 Los Alamos Database (“LANL”, www.hiv.lanl.gov). Subtyping of the clinical strains was initially inferred using a maximum likelihood (ML) phylogenetic tree constructed with general time reversal substitution model with inverse gamma distribution (GTR+G+I) and 1000 bootstrapped data sets, using the Molecular Evolutionary Genetics Analysis software version 5 (MEGA 5) [28]. The gene segments showing clustering with different subtypes for a patient were further used for recombinant screening in Recombination Identification program version 3 (RIP 3.0). To confirm consistent results, REGA HIV-1 Subtyping Tool - version 2.0 was also used.

### Detection of Recombination Events

Different gene segments from an individual showing different subtype clusters were used for the recombination screening. BLAST analysis was performed to identify the nearest sequences using the HIV BLAST tool found in LANL. Reference sequences with nucleotide identity >95% were chosen for each of the clinical strains along with previously determined recombinant strains BC and A1C where all the three segments were available. Separate ML trees were constructed using each of the gene segments. All the clinical strains contributed equally to this analysis and no identical reference sequences were included. The sequences which showed recombination were further processed for detailed analysis of recombination breakpoints in Simplot version 3.5.1 [23]. The mosaic pattern of each unique recombinant form (URF) was confirmed using phylogenetic analysis of the recombination fragments.

### Selection of Reference Strains for tMRCA Analysis

Reference sequences were selected among the full-length subtype C sequences available at the Los Alamos database, since they covered the *env*, *gag* and *pol* regions investigated in this study. Eleven Indian subtype C sequences with a known sampling year available in the LANL pre-made alignment of full-length sequences were also included. In addition, subtype C sequences from this alignment were selected so that all unique combinations of different countries and sampling years were represented once, in order to maintain as much genetic diversity as possible (n = 45). These strains originated from a total of 20 different countries, located in southern Africa (Botswana, Malawi, South Africa, Zambia, N = 18), eastern Africa (Ethiopia, Kenya, Somalia and Tanzania, N = 9); and rest of the world (Argentina, Brazil, Cyprus, Denmark, Germany, Israel, Myanmar, Senegal, Spain, Uruguay, USA and Yemen, n = 18). All the selected reference HIV-1C sequences included in the analysis had the three genes of interest. Two reference subtype B strains were included as out-group. The final data sets, after sequence cleansing and gap stripping, included 196 taxa, 345 sites (*gag*), 159 taxa, 654 sites (*pol*), and 188 taxa, 407 sites (*env*), respectively.

### Estimating Time to the Most Recent Common Ancestors (tMRCA)

Alignments were performed in ClustalX2 [29] and phylogenetic analyses were performed in BEAST v.1.6.2 [20]. Analysis in jModelTest [30] showed that the GTR substitution model with inverse gamma distribution was the best fitting model for all three data sets and it was used in all BEAST runs. Two molecular clock models (‘Relaxed: exponential’ and ‘Relaxed: log-normal’) were tested in combination with four different coalescent tree priors (‘Constant Size’; ‘Exponential Growth’, ‘Logistic Growth’ and ‘Bayesian Skyline’). The resulting log-files were analyzed in Tracer v.1.6.2 [20] and the Bayes Factor analysis showed that the relaxed log-normal clock with Bayesian Skyline was the most appropriate model for all three genes. The taxon sets analyzed were “Subtype C”, which included all subtype C strains, and “India C”, which included all Indian strains from this study. Tip dates (year of sampling for each sequence) together with a previous estimate of the age of subtype C into the year 1952 [10] were used for calibration of the molecular clock (the prior was set to Normal distribution, 49 +/− 6 years since the last year of sampling, 2011), and rates of evolution were automatically calculated from these data. The analyses were run for 100 million generations, with sampling every 10,000 generation, and the sampled trees were annotated using TreeAnnotator v1.6.2 and visualized in FigTree v1.3.1 (http://tree.bio.ed.ac.uk/software/figtree/).
